# Increased firing rates monotonically expand neuronal coding bandwidth

**DOI:** 10.1007/s00422-026-01063-3

**Published:** 2026-09-17

**Authors:** Alexander Wendt, Kolja Klett, Jan Benda, Benjamin Lindner, Jan Grewe

**Affiliations:** 1 Neuroethology, University of Tübingen, Auf der Morgenstelle 28, 72076 Tübingen, Germany; 2 Physics Department, Humboldt University Berlin, Newtonstr. 15, 12489 Berlin, Germany; 3 Bernstein Center for Computational Neuroscience Berlin, Philippstr. 13, Haus 2, 10115 Berlin, Germany; 4 Bernstein Center for Computational Neuroscience Tübingen, Maria-von-Linden-Straße 6, 72076 Tübingen, Germany

**Keywords:** Spiking neurons, LIF model, Encoding, Bandwidth, Coherence

## Abstract

Sensory information is often processed by populations of neurons that vary with respect to their levels of spiking activity. It appears plausible that neurons that fire more often are capable of following faster changes in the input signal than those with lower firing rates. In this study, we test this intuitive assumption by systematically investigating how the spiking activity of a neuron affects information transmission. To this end, we employ the coherence function as a spectral measure of information transmission and study the relation between the width of the coherence function, which we call coding bandwidth, and the firing rate. The reexamination of experimental data from a previous study on weakly-electric fish and the simulation of biologically inspired neuron models fitted to real neurons reveal indeed a significant correlation between firing rate and coding bandwidth. In addition, known analytical expressions for the coherence function for the stochastic leaky integrate-and-fire model demonstrate a linear relation for high firing rates. However, in contrast to intuition, the width of the coherence function becomes independent of the firing rate and is completely set by the membrane time constant for very low firing rates.

## Introduction

Energy is a limiting resource and neuronal signaling is energetically expensive (Attwell and Laughlin 2001; Niven 2016). Accordingly, neural systems need to balance information gain against energy consumption to send only what is needed for a required task (Schreiber et al. 2002). This energy constraint on neural signaling is one of the principles of neural design (Niven et al. 2007; Sterling and Laughlin 2015; Sengupta et al. 2013). The information content of neuronal signals can be quantified by information theoretical measures such as the mutual information (Shannon 1948). The mutual information rate quantifies how much of the information carried by an input signal is still contained in the output of said neuron in bit/s. While obtaining the exact mutual information rate is often unfeasible, for a Gaussian signal, a lower bound of the mutual information can be estimated from spectral measures such as the stimulus–response coherence function (Borst and Theunissen 1999; Rieke et al. 1996; Gabbiani 1996; Bendat and Piersol 2011). It characterizes the contribution of different spectral bands to the overall information transmission and thus provides insights into how the neuron or neural system acts as an information filter. Different cellular and network mechanisms exist for information low-pass, band-pass, and high-pass filtering (Lindner 2016). These filter properties should be distinguished from the more popular concept of power filtering that is solely characterized by a gain or transmission factor (also called susceptibility): information filtering is substantially shaped by both, the transmission of signal power and the power of the background noise and may thus be very different from power filtering. For instance, the cutoff frequency of the gain of the exponential integrate-and-fire neuron model grows linearly with the firing rate (Fourcaud-Trocmé et al. 2003) but it is not clear if this relation also holds for the coherence function. For neurons that act as low-pass filters of information (with a maximum coherence at zero or small frequencies), the coding bandwidth or information-cutoff frequency tells us about the range of frequencies with substantial coherence values.

The leaky integrate-and-fire (LIF) model with white Gaussian background noise and a current stimulus signal allows for the determination of the information-cutoff frequency from analytical expressions. For this model, the coherence function can be calculated based on known expressions for the transfer function (Brunel et al. 2001; Lindner and Schimansky-Geier 2001; Fourcaud and Brunel 2002) and power spectrum (Lindner 2002; Lindner et al. 2002).

An experimental model system in which signal encoding by means of the coherence function has been extensively studied is the electro-sensory system of the weakly electric fish (Krahe and Maler 2014; Grewe 2020). In particular, in wave-type fish electroreceptive neurons, so-called P-units, have been investigated (Chacron et al. 2000, 2001; Barayeu et al. 2023; Grewe et al. 2017; Sharafi et al. 2013). They encode amplitude modulations resulting from interactions of their self-generated quasi-sinusoidal, electric field with the environment, prey, or conspecifics in modulations of their firing rate (Bastian 1981; Nelson et al. 1997; Benda et al. 2005; Barayeu et al. 2023). While prey and navigation signals result in low stimulus frequencies (<20 Hz), amplitude modulations induced by communication signals cover much higher frequencies (200–400 Hz), especially in the context of opposite sex interactions (Henninger et al. 2018).

P-units are quite heterogeneous in various response properties including their baseline firing rate which ranges from 50–400 Hz in *Apteronotus leptorhynchus* (Grewe et al. 2017; Hladnik and Grewe 2023). P-units in *Eigenmannia* have been shown to increase coding fraction and information rate and shorten integration time with higher firing rates (Wessel et al. 1996), already hinting at the dependence of coding bandwidth on firing rate we want to study here.

**Fig. 1:**
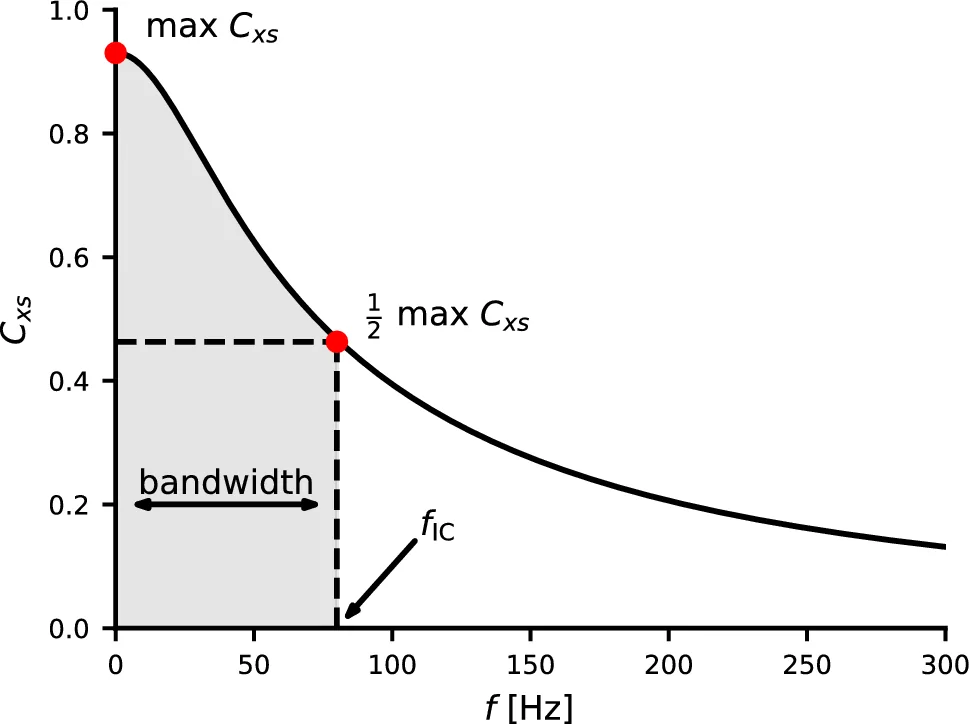
Example coherence function, Eq. (1), of a stochastic LIF model, Eq. (6). We estimate the information-cutoff frequency (ICF, denoted as $f_{IC}$ in figures for brevity), Eq. (2), as a proxy of the coding bandwidth. The ICF is defined as the frequency of the half-maximum of the coherence

In this paper, we analyze the coding bandwidth of single neurons and its dependence on the neuron’s baseline firing rate. We explore this dependence in previously recorded experimental data of P-units, by simulating a LIF-based model of P-units, and by means of an analytical solutions of a stochastic LIF model. In particular, we focus on the information-cutoff frequency (ICF), that we define here as the cutoff of the coherence function at half-maximum (Fig. 1).

## Statistical measures, experimental data, and models

A neuron that is subjected to an external time-dependent stimulus *s*(*t*) encodes this signal in its spike train *x*(*t*). To quantify the neuronal coding bandwidth contained in the spike train about the signal in a frequency-resolved manner, we use the coherence function

1 $$\begin{aligned} C_{xs}(f) = \frac{|S_{xs}(f)|^2}{S_{xx}(f)S_{ss}(f)} \end{aligned}$$

(Borst and Theunissen 1999; Bendat and Piersol 2011; Lindner 2016). Here, $S_{xs}(f)$ is the cross-spectrum of the spike train and the stimulus, while $S_{xx}(f)$ and $S_{ss}(f)$ are the power spectra of the spike train and the stimulus, respectively. The coherence function is a spectral measure of the (linear) information transmission of the neuron. At every frequency, it can be regarded as a squared Pearson correlation coefficient between the input and the output at this frequency component, and it is thus limited to the range $C_{xs}(f) \in [0,1]$ by construction. The coherence estimates how much of the fluctuations of the signal are present in the spike train.

We characterize the width of the coherence function, i.e. the frequency band over which the neuron can transmit significant information (coding bandwidth), by the information-cutoff frequency (ICF) that we define as the frequency at which the coherence attains half of its maximum value:

2 $$\begin{aligned} C_{xs}(f_{\text {IC}}) = \frac{1}{2}\max \limits _{f}\left[ C_{xs}(f)\right] . \end{aligned}$$

The definition of the ICF is illustrated in Fig. 1, for a low-pass coherence function with a maximum at zero frequency. In equations and figures we denote the ICF as $f_{\text {IC}}$.

This simple definition of the coding bandwidth suffices for the mostly low-pass coherence functions we investigate here. While we also observe some coherence functions with band-pass character, their maxima are at small non-vanishing frequencies, such that the information-cutoff frequency can be interpreted as the stimulus encoding bandwidth. Even for coherence functions that have a pronounced band-pass character, i.e. a maximum at large non-vanishing frequencies, we can still apply the information-cutoff frequency as a proxy of the upper limit of the stimulus encoding band-width. We also put forward alternative formulations of the coding bandwidth that might be better suited for more complex shaped coherence functions, see disscussion 4.8.

The coherence function is related to the mutual information rate (MIR). For a Gaussian signal, a lower-bound of the MIR can be estimated from the integral of the logarithmically transformed coherence function (Rieke et al. 1993, 1996; Gabbiani 1996)

3 $$\begin{aligned} R_\text {Info, LB} = - \int _0^\infty \textrm{d}f\, \log _{2}\left[ 1-C_{xs}(f)\right] . \end{aligned}$$

Here, however, we do not consider this measure of information transmission as the integration does not preserve information about which frequencies are transmitted.

### Leaky integrate-and-fire model

In the leaky integrate-and-fire (LIF) model, the dynamics of the sub-threshold membrane potential of the neuron *v* is governed by a stochastic differential equation

4 $$\begin{aligned} \tau _m \dot{v} = -v + \mu +\xi (t) + s(t), \end{aligned}$$

where $\tau _m$ is the membrane time constant, μ is the offset the neuron receives, ξ(t) is a zero-mean noise, and *s*(*t*) is an additional signal that drives the neuron. In addition, the membrane potential is subject to a fire-and-reset rule. When the membrane potential reaches a threshold value $v_T$, a spike is generated and the potential is instantaneously reset to a fixed value $v_R$. Following an absolute refractory period $\tau _\text {ref}$, the dynamics of the voltage are resumed. The spike train, produced in this manner, is a sum of Dirac delta functions centered at the spike times ${t_i}$:

5 $$\begin{aligned} x(t) = \sum _{\{t_i\}}\delta (t-t_i). \end{aligned}$$

In Eq. (4) we measure the membrane potential in multiples of the threshold-reset distance and with respect to the reset voltage as a reference point; this choice implies $v_T$ and $v_R$ being set to unity and zero, respectively.

To apply existing analytical results, we set ξ and *s* as two independent parts of a total white Gaussian noise input to the neuron (Vilela and Lindner 2009). In other words, we consider the neuron to be subject to a white Gaussian noise and interpret a fraction of the noise as the external stimulus. Then, Eq. (4) reads

6 $$\begin{aligned} \tau _m \dot{v} = -v + \mu +\sqrt{2D\tau _m}\left[ \sqrt{1-c}\,\xi _i(t) + \sqrt{c}\, \xi _s(t)\right] , \end{aligned}$$

with c∈[0,1] setting the relative intensity of the internal noise $\xi _i(t)$ and the external noise $\xi _s(t)$, where both have a delta correlation $\langle \xi _{i}(t)\xi _{i}(t')\rangle = \langle \xi _{s}(t)\xi _{s}(t')\rangle = \delta (t-t')$. Constructing the LIF model in this way provides an exact expression for the coherence function for all c∈[0,1], i.e. also for strong stimuli beyond the linear regime (Vilela and Lindner 2009).

#### Analytical result for the coherence function

We obtain an analytical expression of the coherence function $C_{xs}$ by introducing response statistics of the firing rate into Eq. (1) and making use of known results for the power spectrum and susceptibility for the LIF model.

First, we note that for the white-noise signal we consider here ($s(t) = \sqrt{2cD\tau _m}\xi _{s}(t)$ with $S_{\xi _s\xi _s}(f) = 1$), the power spectrum of the stimulus is $S_{ss}(f) = 2cD\tau _m$ and the cross-spectrum $S_{xs}(f) = \sqrt{2cD\tau _m} S_{x\xi _s}(f)$. Then, the coherence function (Eq. (1)) reduces to just the ratio of squared cross-spectrum and spike-train power spectrum:

7 $$\begin{aligned} C_{xs}(f) = \frac{|S_{x\xi _s}(f)|^2}{S_{xx}(f)}. \end{aligned}$$

Applying the Furutsu-Novikov theorem (Furutsu 1963; Novikov 1965) to the cross-spectrum (see Lindner 2022; for further details, see appendix of Klett and Lindner 2025) yields

8 $$\begin{aligned} S_{x\xi _s}(f) = \sqrt{2cD\tau _m}\;\chi _x(f), \end{aligned}$$

where the susceptibility $\chi _x$ describes the time-dependent firing rate *r*(*t*) in response to a signal in frequency space via $\tilde{r}(f) = \chi _x(f)\tilde{s}(f)$. The coherence function then reads

9 $$\begin{aligned} C_{xs}(f) = \frac{2cD\tau _m|\chi _x(f)|^2}{S_{xx}(f)}. \end{aligned}$$

For the LIF model with white noise, the two remaining statistics on the right are known analytically: the spike-train power spectrum (Lindner et al. 2002; Lindner 2002)

10 $$\begin{aligned} \begin{aligned}&S_{xx}(f) = \tau _mr_{0} \\&\quad \frac{\big |\mathcal {D}_{i2\pi f \tau _m}(z_T)\big |^2 - \big |\textrm{e}^{\frac{z_R^2-z_T^2}{4}}\mathcal {D}_{i2\pi f \tau _m}(z_R)\big |^2}{\big |\mathcal {D}_{i2\pi f \tau _m}(z_T) -\textrm{e}^{i2\pi f \tau _\text {ref}}\textrm{e}^{\frac{z_R^2-z_T^2}{4}}\mathcal {D}_{i2\pi f \tau _m}(z_R)\big |^2}, \end{aligned} \end{aligned}$$

and the spike-train susceptibility (Brunel et al. 2001; Lindner and Schimansky-Geier 2001)

11 $$\begin{aligned} \begin{aligned}&\chi _x(f) = \frac{2 \pi f\tau _mr_{0}}{\sqrt{D}(2\pi f \tau _m + i)}\\&\quad \frac{\mathcal {D}_{i2\pi f \tau _m - 1}(z_T) - \textrm{e}^{\frac{z_R^2-z_T^2}{4}}\mathcal {D}_{i2\pi f \tau _m - 1}(z_R)}{\mathcal {D}_{i2\pi f \tau _m}(z_T) - \textrm{e}^{i2\pi f \tau _\text {ref}}\textrm{e}^{\frac{z_R^2-z_T^2}{4}}\mathcal {D}_{i2\pi f \tau _m}(z_R)}, \end{aligned} \end{aligned}$$

where $\mathcal {D}_{a}(z)$ denotes the parabolic cylinder function (Abramowitz and Stegun 1972) with arguments $z_{T} = (\mu - v_{T})/ \sqrt{D}$ and $z_{R} = (\mu - v_{R})/ \sqrt{D}$. $r_0$ is the stationary firing rate, which is independent of *c* and thus also coincides with the baseline firing rate obtained without external stimulation (c=0). Inserting Eqs. (10) and (11) into Eq. (9) gives an relation for the coherence function in terms of parabolic cylinder functions

12 $$\begin{aligned} \begin{aligned}&C_{xs}(f) = \frac{8\pi ^2c\tau _m^3r_0f^2}{1+(2\pi f \tau _m)^2}\\&\quad \frac{|\textrm{e}^{z_T^2/4}\mathcal {D}_{i2\pi f \tau _m-1}(z_T)-\textrm{e}^{z_R^2/4} \mathcal {D}_{i2\pi f \tau _m-1}(z_R)|^2}{|\textrm{e}^{z_T^2/4}\mathcal {D}_{i2\pi f \tau _m}(z_T)|^2-|\textrm{e}^{z_R^2/4} \mathcal {D}_{i2\pi f \tau _m}(z_R)|^2}. \end{aligned} \end{aligned}$$

This is a closed analytical expression for $C_{xs}(f)$ as the spontaneous firing rate is given by (Ricciardi 1977; Brunel and Hakim 1999)

13 $$\begin{aligned} r_0 =\,&\left[ \tau _\text {ref}+\tau _m\sqrt{\frac{\pi }{2}}\int _{z_T}^{z_R}\textrm{d} z\, \textrm{e}^{z^2/2} \textrm{erfc}\left( z/\sqrt{2}\right) \right] ^{-1}\nonumber \\ =\,&\left[ \tau _\text {ref}{+} \tau _m\int _{0}^{\infty }\textrm{d} u\, u^{-1} \textrm{e}^{-u^2} \left( \textrm{e}^{-\sqrt{2}z_T u}{ -} \textrm{e}^{-\sqrt{2}z_R u}\right) \right] ^{-1}. \end{aligned}$$

Notably, $C_{xs}$ does only depend on the absolute refractory period via $\tau _\text {ref}$ appearing in Eq. (13). The contributions in Eqs. (10) and (11) cancel. Since $r_0$ is only a constant factor in Eq. (12) that does not affect the frequency dependence, the ICF of the coherence function is independent of $\tau _\text {ref}$. The same arguments also apply to *c*. The shape of the coherence function and its ICF are set by the total noise in the system and thus are independent of the stimulus fraction *c* of the total noise. Thus, we set c=0.1 and, for the analytical part of this study, $\tau _\text {ref} = 0$. Moreover, the membrane time constant simply scales the frequency axis, since it appears only in products with $f/r_0$ in Eq. (12) (if $\tau _\text {ref} = 0$). If we fix the membrane time constant, the coherence function and the firing rate are solely determined by the two parameters of the LIF model μ and *D*. So, we obtain the relation between ICF and firing rate by evaluating Eqs. (12) and (13) for various (μ,D).

### P-unit model

The P-unit model extends the LIF model, Eq. (4), to reproduce signal processing at the different stages in electrorecptor neurons and their afferents (Barayeu et al. 2026a). These include rectification of the input signal at the first synapse, dendritic low-pass filtering and adaptation currents.

First, we approximate the fish’s self-generated electric organ discharge (EOD) as a sinusoidal waveform with a specific the EOD frequency ($f_{\text {EOD}}$) and an amplitude of one:

14 $$\begin{aligned} y(t) = \sin (2\pi f_{\text {EOD}} t). \end{aligned}$$

The EOD acts as a carrier signal and is, under baseline conditions, the sole external input to the P-unit model. The neurons respond to amplitude modulations of the self-generated EOD and to stimulate the model with Gaussian white-noise amplitude modulations *s*(*t*), the baseline EOD is multiplied by 1+αs(t), where the contrast α sets the amplitude of the noise stimulus *s*(*t*) relative to the amplitude of the EOD *y*(*t*). The model input *u*(*t*) is thus given by:

15 $$\begin{aligned} u(t) = (1+\alpha s(t))y(t). \end{aligned}$$

*u*(*t*) is then rectified, representative of the chemical synapse between the primary receptor cell and the P-unit afferent, and low-pass filtered, time constant $\tau _{d}$ subsuming all low-pass properties of passive conduction in dendrites and synaptic dynamics, yielding the dendritic membrane potential $v_{d}$:

16 $$\begin{aligned} \tau _{d} \dot{v}_{d} = -v_{d}+ \lfloor u(t) \rfloor _{0}. \end{aligned}$$

where

17 $$\begin{aligned} \lfloor u(t) \rfloor _{0} = \left\{ \begin{array}{ccl} u(t) & , & u(t) > 0 \\ 0 & , & \text {else} \end{array} \right. \end{aligned}$$

denotes thresholding at zero.

The resulting voltage is multiplied by a factor β, that adjusts the cell’s sensitivity for the driving input, when it enters the LIF equation for the membrane potential $v_m(t)$ as its input:

18 $$\begin{aligned} \tau _{m} \dot{v}_{m} = - v_{m} + \mu + \beta v_{d} - a + \sqrt{2D}\xi (t). \end{aligned}$$

Here, $\tau _{m}$ is the membrane time constant, μ is an offset, and $\sqrt{2D}\xi (t)$ is an intrinsic white-noise. The adaption current *a* is subtracted from the membrane voltage and decays exponentially to zero according to

19 $$\begin{aligned} \tau _{a} \dot{a}= - a. \end{aligned}$$

with adaptation time constant $\tau _{a}$ (Benda and Herz 2003; Fisch et al. 2012). Whenever the membrane voltage crosses the threshold $v_T = 1$, a spike is registered, $v_m$ is set to $v_R = 0$, and the adaption variable is incremented by Δa:

20 $$\begin{aligned} v_m(t) \ge v_T \; : \left\{ \begin{array}{rcl} v_m & \mapsto & 0 \\ a & \mapsto & a + \Delta a/\tau _a \end{array} \right. . \end{aligned}$$

Lastly, the model contains a refractory period, during which the integration of the membrane voltage $v_m(t)$ is paused after spiking.

#### Fitting process

The parameters of the P-unit models were fitted to experimental data in the context of a previous project, and were shown to accurately reproduce the cellular response characteristics (see (Barayeu et al. 2023, 2026a, b)). The model, python code, and the parameterization of the 39 model cells are freely available https://github.com/bendalab/punitmodel. In brief: From our pool of recorded data, P-unit recordings were selected in which the spontaneous baseline activity and the responses to step stimuli were recorded. The parameters of the P-unit model were then fitted to individual experimentally characterized neurons applying a simplex fit approach. The error function was based on the mean-square-errors between the model and the real cell’s baseline characteristics and responses to step stimuli. The spontaneous baseline activity was characterized by the firing rate, histogram of the interspike intervals (ISI), serial-correlation of the ISIs, and coupling to the underlying EOD carrier. The responses to steps in stimulus intensity were characterized by the onset and the steady-state firing rates. **Note:** The population of cells selected for model fitting has only very small overlap (two cells) with the selection of cells that were used for the coherence analysis done in Sect. 2.3. For the latter we needed recordings that included responses to the same frozen band-limited noise stimulus. Distributions of model parameters for the 39 model neurons are shown in Fig. 7.

#### Simulation parameter

For each model cell, we simulated 10 000 trials with a duration of 100 s and a sampling frequency of 100 kHz. Of each trial, only the last $2^{22}$ data points were used for further analysis. Spike train power spectra $S_{xx}(f)$, stimulus spectra $S_{ss}(f)$, and the cross spectra $S_{xs}(f)$ were estimated with a frequency resolution of 0.029 Hz. The stimulus contrast α was set to 10 % and the white-noise amplitude modulations had a cutoff frequency at 300 Hz. Spectra were averaged over segments, and the coherence and its ICF were calculated in Eqs. (1) and (2), respectively.

### Experimental recordings from P-units

The experimental data consist of single cell recordings of P-units, previously published in Grewe et al. (2017) where the data is publicly available (https://doi.gin.g-node.org/10.12751/g-node.5b08du). From this dataset, we selected P-units that were stimulated with a band-limited Gaussian white noise stimulus with a cutoff frequency of 300 Hz and a contrast of 10 %. Experimental data was recorded with a sampling rate of 20 kHz. For each P-unit we selected ten trials, and inspected each trial for consistent spiking. This selection process resulted in 51 cells that were used for further analysis.

To estimate the coherence function, Eq. (1), the average spike train power spectra $S_{xx}(f)$, stimulus power spectra $S_{ss}(f)$ and the cross power spectra $S_{xs}(f)$, were computed as described in Sect. 2.2. This estimation was based on segments of $2^{13}$ data points. At each segment the discrete Fourier transformation was calculated, which resulted in a frequency resolution of 2.44 Hz. The coherence function was afterwards smoothed by a mean filter with a length of 5 data points for a more robust estimation of the ICF (Eq. (2)).

To compare the different models and experimental data we also computed the coefficient of variation (*CV*) of the interspike intervals during baseline activity (α=0 in Eq. (15)), where only the fish’s own EOD, Eq. (14), was present.

## Results

To investigate the relation between firing rate and information-cutoff frequency (ICF) of the coherence function, we first re-analyzed the experimental data from P-units, then compared these findings to P-unit model simulations as well as to analytic solutions of the stochastic LIF model.

**Fig. 2:**
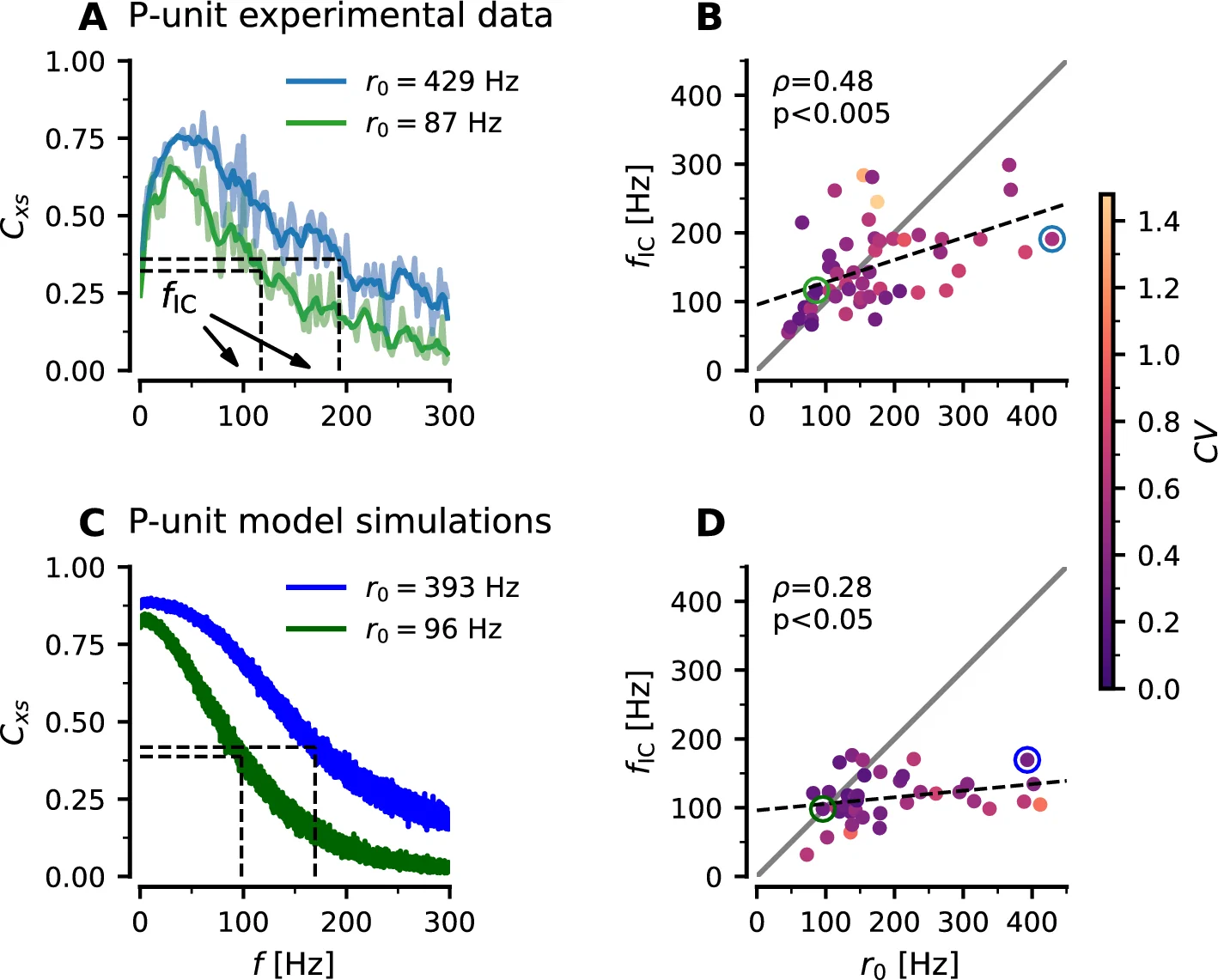
Information-cutoff frequency increases with baseline firing rate of experimentally recorded P-units (**A, B**) and simulated P-unit neuron models (**C, D**). Left: Examples of coherence functions from experimental recordings (**A**) and model neurons (**C**) with low (green) and high (blue) firing rates. In **A** shaded colors depict the original data, and in opaque colors are the smoothed (mean filter with 5 data points) coherence functions. Right: Scatter plot of the cutoff frequency, Eq. (2), and the respective firing rate of the experimental (**B**, n=51) and simulated data (**D**, n=39). Circles around scatter dots indicate the cells for which the coherence function is shown in A/C. The gray line is the identity line. A linear regression was fitted to the data points (black dashed line). The color encodes the CV of the individual cells under baseline conditions

### Information-cutoff frequency increases with firing rate in experimental data

The spontaneous activity of P-units varies in a wide range from 50 up to 400 Hz (Grewe et al. 2017; Hladnik and Grewe 2023). These large differences in firing rate influence the encoding of amplitude modulations of their self-generated electric field. This is exemplified by the coherence functions of two example neurons in Fig. 2A. The neuron with a high firing rate of $r_0=429$ Hz (blue) has an overall higher coherence and encodes over a wider range than the example neuron with a lower baseline firing rate of $r_0=87$ Hz (green, Fig. 2A). Indeed, the ICF of the coherence function measured at half-maximum coherence is about 75 Hz higher in the neuron with the high firing rate. This increase, however, is much less than the difference in baseline firing rates of more than 300 Hz.

Across all P-unit recordings, we find a significant positive correlation between the firing rate and the ICF, demonstrating that neurons with higher firing rates are better in conveying high-frequency information than neurons with a lower firing rate (Pearson ρ=0.48, p<0.005, Fig. 2B). Neurons within the lower regime of baseline firing rates (50–200 Hz) gather along the identity line between cutoff frequencies and firing rates. Observed ICFs increasingly spread with higher firing rates. Among P-units with higher baseline firing rates (>200 Hz) the cutoff frequencies still increase but do not keep up with firing rates (slope of regression line is 0.33). Instead, the cutoff frequencies stay below the corresponding firing rates.

### LIF-based P-unit model reproduces experimental observations

To further confirm these experimental observations, we also analyzed responses from physiologically inspired LIF models that were fitted to specific electrophysiologically measured P-units (see methods). The P-unit model neurons (N=39) have similar responses and coherence functions (Fig. 2C) as the experimentally measured ones. An example model neuron with a high firing rate of $r_0=393$ Hz (blue) also shows overall higher coherence values than a model neuron with a low baseline firing rate of $r_0=96$ Hz (green). The ICF of the high firing rate model neuron is 70 Hz higher than that of the low firing rate neuron.

Note that the coherence functions clearly differ between model cells and experimental data. In the experimental data, the coherence functions drop at lower frequencies (<50 Hz) whereas in the model neurons they stay at high values. This can be attributed to a finite size effect of the limited experimental data where the number of segments used for the Fourier transformations is too low to properly estimate the spectral components in the low-frequency range (see appendix 1).

The information-cutoff frequencies of the P-unit models also increase with higher baseline firing rates (Pearson ρ=0.27, p=0.046, slope of regression is 0.1), but weaker than in the experimental data (Fig. 2D). ICFs at similar firing rates tend to be lower in the models than in the experimental data. We further observe that the coefficient of variation of the interspike intervals, *CV*, a proxy of the intrinsic noise strength, is positively correlated with the firing rate (Pearson ρ=0.39, p=0.0067 in model cells, ρ=0.34, p=0.0066 in experimental data). In the models this can be explained by higher levels of intrinsic noise (parameter *D*). In the experimental data we observe that cells with higher firing rates tend to show bursts of action potentials. The fraction of spikes within burst packages on the total spike count is clearly correlated with an increased *CV* (Barayeu et al. 2024). Note, that in the experimental data the *CV*s (0.52±0.25) are on average higher than in the simulated model neurons (0.42±0.21, mean±SD, Mann–Whitney U test: U=718.0, p=0.025), because the cells used for fitting the models are biased towards non-bursting cells.

Here we showed that information-cutoff frequencies of p-type electrosensory afferents increase with baseline firing rates. This indicates that P-units with higher firing rates are better in conveying information about higher stimulus frequencies. To further examine this relationship we next consider a stochastic LIF model.

### Relation between information bandwidth and firing rate for a stochastic LIF model

In contrast to the extended P-unit model, an analytical solution for the coherence function of the stochastic LIF model, Eq. (12), exists. This allows for a straightforward determination of the ICF without actual numerical simulations of the LIF model. Complete control over all model parameters enables us also to test whether the relation between the ICF and the firing rate is exclusive to P-units, a special kind of high-activity neurons, or can also be found in more generic neurons. In our model, we distinguish the case of a high-activity P-unit-like neuron from the case of a more generic neuron, for instance from cortex, based on the membrane time constant.

**Fig. 3:**
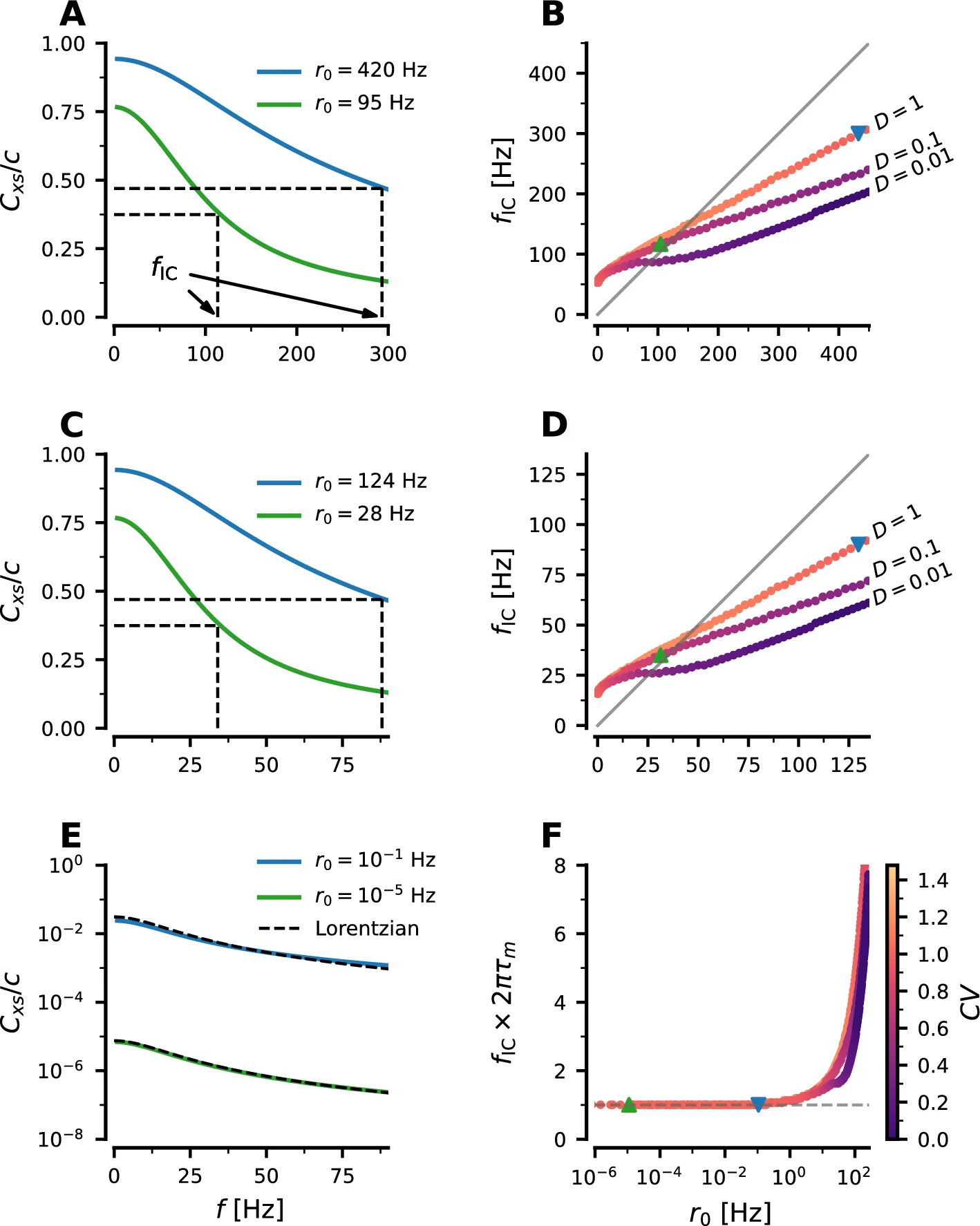
Numerical evaluation of the analytical result for the coherence function of the LIF model with white noise Eq. (12). **A** Example of two coherence functions for $\tau _m =$ 3 ms representative of P-units with a high firing rate (blue) and a low firing rate (green). **B** Cutoff frequency of the coherence function for varying firing rates for $\tau _m =$ 3 ms. The solid line indicates the identity function. The noise level is denoted at the corresponding data series. The position of example coherence functions depicted in A are highlighted by the green and blue triangles. **C** Example of two coherence functions for $\tau _m =$ 10 ms representative of neurons in cortex (Koch et al. 1996) with a high firing rate (blue) and a low firing rate (green). Green and blue curves in A and C are for the same μ and *D*. Note that the frequency axis in C is rescaled compared to A reflecting the larger $\tau _m$. **D** Cutoff frequency of the coherence function for varying firing rates for $\tau _m =$ 10 ms. The solid line indicates the identity function. The position of example coherence functions shown in C are indicated by triangles. Note: both frequency axes have been rescaled from B to D due to change in $\tau _m$ leading to same curves in new coordinates. **E** Example of two coherence functions very low firing rates (solid lines) compared with their respective Lorentzian asymptotics from Eq.(12) (dashed lines). **F** Cutoff frequencies from D relative to the rare-firing limit Eq. (22) for logarithmic firing-rate axis. Dashed line indicates unity. Data points in B, D and E are colored according to the *CV* that corresponds to the chosen (μ, *D*), for details see Lindner et al. (2002). For all plots $\tau _\text {ref} = 0$

We obtain the information-cutoff frequency (ICF) for a fixed value of the membrane time constant by evaluating Eq. (12) for various intensities of the white noise and mean inputs. Namely, $D \in \{0.01, 0.1,1\}$, and for each *D* we control the firing rate by tuning through μ. The firing rate is calculated using Eq. (13). For better comparison with the P-unit data, we also color the data points according to their *CV* (Fig. 3).

We start out with a small value of the membrane time constant, $\tau _m =3$ ms, corresponding to a high-activity neuron, such as the P-unit (Chacron et al. 2000), and inline with the values used in the P-unit model presented in Sect. 2.2. Figure 3A shows that the coherence function for a high firing rate (blue) exceeds that of a low firing rate (green) systematically. More importantly for our study, the coherence function for a higher firing rate decays slower leading to an increased ICF compared to the case of a lower firing rate.

Note, that in our LIF model, *D* scales the total noise in the system (stimulus + intrinsic noise) and that the shape of the coherence function and thus the ICF depend on this total noise. How much of this total noise is considered the stimulus is determined by the stimulus fraction *c*. Hence, a fixed value of *D* can correspond to a neuron with lots of intrinsic noise and a minute noise stimulus or even a noiseless neuron with a sufficiently strong stimulus depending on *c*. However, *c* has no effect on the shape of the coherence function and the ICF. It enters only as a constant in Eq. (12). To restore the familiar [0, 1] range of the coherence function, the coherence functions shown here are divided by *c*.

The ICFs for a wide range of firing rates is shown in Fig. 3B. For firing rates above 150 Hz ($\approx 0.5 \tau _m^{-1}$), we see a clear trend: the information-cutoff frequency grows with the firing rate but is smaller than it. In particular, we find for the LIF model that the ICF increases approximately linearly with $r_0$ at high firing rates. The corresponding regression slopes are ≈0.53 (D=1), ≈0.41 (D=0.1) and ≈0.43 (D=0.01), are smaller than one.

There also appears to be a monotonic relation between the *CV* and ICF: $f_\textrm{IC}$ increases with the *CV* for a fixed $r_0$. This is contrary to the maximum coherence values at f=0 of the LIF model, which depends non-monotonically on the *CV* (Fig. 6). It is difficult to tell whether the *CV* has the same effect in P-units due irregular distribution of observed firing rates (Fig. 2B, C).

For firing rates around 100 Hz ($\approx 0.3 \tau _m^{-1}$), the exact value of the rate depends on the *CV*, the ICF is close to the firing rate as also seen for P-units. Also, in this frequency range we observe a saddle point in the curve with the lowest *CV* where the ICF remains constant. The other two curves display strictly monotonic growth.

For firing rates less than 50 Hz ($\approx 0.15 \tau _m^{-1}$), all curves converge to the same point. This is not observed in the P-unit data as all the cells studied display firing rates above 50 Hz. We further analyze this limiting behavior at low firing rates later.

In the LIF model, the membrane time constant simply scales time and frequency with $\tau _m$ and $1/\tau _m$, respectively. We illustrate this effect of the membrane time constant for $\tau _m =$ 10 ms (Fig. 3C), reflecting the larger membrane time constants typical for cortical neurons firing at low rates (Koch et al. 1996; Bastian et al. 2002).

The example coherence functions in Fig. 3C are based on the same μ and *D* values as the ones used in Fig. 3A. Only the frequency axis and the firing rates are rescaled due to the change in $\tau _m$.

The ICF is affected by $\tau _m$ the same as the frequency. Thus, the relation between the firing rate and the ICF still persists when changing $\tau _m$ (Fig. 3D). The slopes for the different noise levels remain the same. However, the absolute frequencies to which the ICF converges for $r_0 \rightarrow 0$, at which cutoff frequency and firing rate cross, and at which the linear growth of the ICF starts, change due to the rescaling of the axis. Of course, when expressing these frequencies in multiples of $\tau _m$ they do not change with $\tau _m$.

The rare-firing regime appears to contradict the intuitive expectation that higher firing rates lead to better transmission of high frequency stimuli. For $r_0 \rightarrow 0$ the ICFs accumulate independently of μ and *D* at a non-vanishing value far greater than the firing rate. We analyzed this convergence of the ICF by considering the asymptotic behavior of Eq. (12) for $r_0 \rightarrow 0$ (see appendix 1). It turns out that the coherence function attains a $1/(1+x^2)$ (Lorentzian) shape. Namely,

21 $$\begin{aligned} C_{xs}(f)\sim \frac{2cr_0\tau _mz_T^2}{1+(2\pi f \tau _m)^2} . \end{aligned}$$

This coincides with the low-firing-rate asympotics of $|\chi _x(f)|^2$ (Ostojic and Brunel 2011) as $S_{xx}(f)$ becomes constant for rare (Poisson) spiking with a *CV* →1 for $r_0 \rightarrow 0$. Equation (21) provides a good approximation of the coherence function for very low firing rates (Fig. 3E). Especially at low stimulus frequencies, which are relevant for determining the cutoff frequency, the approximate Lorentzian and the exact coherence function match well. Notably, the ICF of Eq. (21) $f_\textrm{IC}^{*}$ depends only on the membrane time constant

22 $$\begin{aligned} f_\textrm{IC}^{*} = \frac{1}{2\pi \tau _m}\approx \text {16 Hz} \end{aligned}$$

as can be seen in Fig. 3F. This is the lowest ICF. Increasing the neuron’s firing rate increases ICF to higher values.

Figure 3E also illustrates the drastic and systematic decrease of the coherence function with vanishing firing rate. So, while the ICF consistently stays above the firing rate for very small $r_0$, the actual information transmission is minuscule. This collapse of the coherence function for rare firing is illustrated in Fig. 6.

## Discussion

We investigated the relationship between the firing rate of a neuron and its linear stimulus encoding performance. In particular, we analyze the information-cutoff frequency (ICF, Eq. (2)) of the stimulus–response coherence in experimentally recorded p-type electroreceptor afferents (P-units) of weakly electric fish, in a phenomenological LIF-based model of such neurons, and in the stochastic LIF model. P-units are heterogeneous in their baseline firing rates (Grewe et al. 2017; Hladnik and Grewe 2023), and neurons with low firing rates have a low ICF while high firing rate neurons have a higher ICF, i.e. a wider coding bandwidth. The same effect could be observed in P-unit model neurons, and in the stochastic LIF model. We leveraged the latter to derive the exact relations between the firing rate and the information-cutoff in the high-firing regime typical of P-units and also in low firing rate regimes that is more realistic e.g. for cortical neurons. Furthermore, we demonstrated that at very low firing rates the ICF is solely determined by the membrane time constant.

### Previous studies show increase in mutual information rate, not information-cutoff frequency

We are not the first to notice that the firing rate has an effect on the stimulus encoding performance. Previous studies, however, focussed on the mutual information not the encoding bandwidth. Haag and Borst (1998), for example show that the mutual information rate, i.e. the integral of the coherence function (3), increases with the firing rate in the lobula-plate tangential cells of the fly visual system.

In the electroreceptors of the related species of weakly electric fish, *Eigenmannia virescens*, a positive correlation between the mean firing rate and the mutual information was described (Wessel et al. 1996). In principle, the mutual information rate can be increased by a higher SNR and/or a higher information bandwidth. There, changes in firing rate of the same neuron were induced by varying the stimulus intensity. The increased firing affects mainly the signal-to-noise ratio (SNR) and not the information-cutoff frequency. Unfortunately, the work does not compare the encoding performance across neurons that have different firing rates under the same stimulus condition.

### Increase of information-cutoff frequency with firing rate

The simulations of the stochastic LIF model clearly show that the higher a neuron’s firing rate the better high-frequency stimulus components can be encoded. The information-cutoff frequencies are at approximately half the firing rate. This makes intuitively sense, because if there are not enough spikes to resolve fast changes in the stimulus the decoder has no way to know what happened in between the spikes.

In the electrophysiologically recorded P-units, the relation between ICF and average firing rate on a first glance looks similar, but the slope of the regression line is smaller than the slopes in the stochastic LIF model. In the P-unit neurons, this slope is even smaller. Why do P-units apparently need more spikes per time to achieve the same ICF than the stochastic LIF model?

Note that we here compare quite different things: In the stochastic LIF model, we fix the model parameters (membrane time constant and noise strength) and systematically change the offset current in order to change the neuron’s firing rate and to observe the corresponding ICF. In case of the P-units, on the other hand, we simply look at the properties of a heterogeneous population of cells. Each cell has a set of parameter values, including membrane time constant, noise strength, and offset current. We can not (in the experiments) or do not (in the simulations) systematically change them, as the parameterization resulted from fits to real neurons. Thus, the regression line in the P-unit data tells us something about the actual distribution of parameter values in the population of recorded cells. By selecting specific combinations of membrane time constants, noise strengths, and offset currents, one can imagine that it is possible to obtain a population of stochastic LIF model neurons that might resemble our findings from the P-units.

In the P-units, however, additional mechanisms are at work that limit the maximum ICF. This we discuss in the following sections. In particular we address the limits imposed on the ICF by the time-constant of the dendritic pre-filter.

### Increase of information-cutoff frequency with smaller membrane time constants

The membrane time constant simply scales times and frequencies in the stochastic LIF model, Eq. (12) for fixed offset current and noise strength. In this setting, the membrane time constant does not change the shape of the coherence function and directly scales the ICF as well (Fig. 3). However, when we ask how the membrane time constant influences the coherence function for a given and fixed firing rate, then the situation is more complicated.

The firing rate is inversely proportional to the membrane time constant when ignoring the refractory period, Eq. (13). Maintaining a certain firing rate, requires to adapt the proportionality factor by means of changing the offset current and noise strength. These two parameters, however, change the shape of the coherence function and thus the ICF. This is the reason why in Fig. 3B we get for each firing rate and a fixed membrane time constant three different values of the ICF, one for each of the three different noise strengths.

When we now increase the membrane time constant at a given firing rate we have to consider both effects: the time constant scales frequencies down but we also have to adjust the offset current and/or noise strength, which in turn also influences the ICF. Comparing panels B and D in Fig. 3 suggests that the ICF is still reduced, but less than the pure scaling effect of the membrane time constant. Viewed the other way around, the membrane time constant is still a major determinant of a ICF at a fixed firing rate of the LIF model. The ICF can be primarily increased by decreasing the membrane time constant, but secondarily also by increasing the noise strength, for example.

### Low-pass pre-filtering imposes an upper limit on the information-cutoff frequency in P-units

In addition to the membrane time-constant, two more time scales affect the stimulus encoding bandwidth. The adaptation time constant, Eq. (19), attenuates low stimulus frequencies in the neuron’s transfer function (Benda and Herz 2003; Benda et al. 2005) but does not influence the coherence (Middleton et al. 2003, see Appendix 1).

**Fig. 4:**
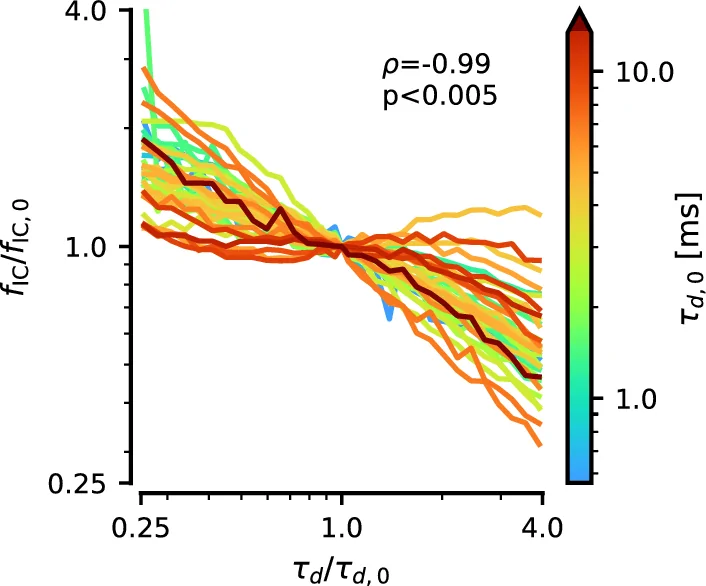
Dendritic low-pass filter constrains ICF. For each model neuron, the dendritic time-constant $\tau _d$ was changed in the range 0.25–4 times its original value ($\tau _{d_0}$) and the resulting ICF, $f_{\text {IC}}$ was normalized to the $f_{\text {IC,0}}$, observed with $\tau _{d,0}$. Line coloring encodes $\tau _{d,0}$ of each model neuron. Numbers are the Pearson correlation coefficient and the respective *p* value

The pre-filtering of the incoming signal after synaptic rectification Eq. (16) before it enters the spike generator is modeled here as a first-order low-pass filter with the time-constant $\tau _d$. This filter represents any low-pass effects due to passive conduction in the electroreceptor dendrites or the low-pass properties of the synapse. The respective time-constant ($\tau _d$ in Eq. (16), median: 3.1 ms, inter-quartile range: 1.5–5.4 ms) is, on average, twice as high as the membrane time-constant ($\tau _m$ in Eq. (18), median: 1.5 ms, inter-quartile range: 1.2–2.1 ms). The larger time-constants of this additional low-pass filter limits the representation of high frequency information that cannot be compensated for by higher firing rates. Indeed, manipulating this time-constant of the P-unit models strongly affects the ICF (Fig. 4). Reducing the $\tau _d$ clearly shifts the ICF to higher frequencies (Pearson ρ=-0.99, p<0.005). All model cells follow the general trend but the details of the dependency on the $\tau _d$ vary across models. Obviously, more than a single factor determines the ICF as we already discussed above.

In the P-unit model, the low-pass pre-filter is required to reduce the spike time locking to the fish’s self generated electric field to the experimentally observed ranges (Sinz et al. 2020). Here, it evidently also impacts the ICF by limiting the spectral content of the signal reaching the spike generator. In the original fitting process, $\tau _d$ was probably not well constrained as the models were not fitted to the noise responses used here. Instead, fitting involved matching characteristics of baseline firing and responses to step stimuli (see methods 2.2.1). Thus, the time-constants of the pre-filter could have been overestimated and this could explain why the ICFs of the P-unit models stay so low and are less dependent on their firing rate (Fig. 2D).

The limiting effect of the dendritic low-pass filter could also explain why the spike-triggered averages (STAs) reported by Wessel et al. (1996) depend so little on the firing rate. Even though they did change the firing rate experimentally, the form of the STA remains almost unchanged. If the encoding bandwidth had changed substantially, the STAs should have become narrower.

### Relevance of high-frequency electrosensory information

Electric fish use their self-generated electric fields for navigation, prey detection, and communication. Interactions between the self-generated field and the surroundings lead to amplitude modulations (AMs) which are encoded in the p-type electroreceptor afferents. In the context of social interactions, the behavioral relevance of high-frequency information was recently highlighted. Male–female courtship interactions create AMs of frequencies exceeding 300 Hz (Henninger et al. 2018).

The results shown here suggest that first of all it would need neurons with high firing rates to encode such behaviorally relevant high-frequency signals. Furthermore, as we just discussed, it requires also a small dendritic time constants. On the level of the afferents in the sensory periphery, it seems plausible that such information can be encoded in a subset of P-units. However, we do not have any evidence for a selective readout of high-firing P-units on the next processing step, the pyramidal cells in medullary electrosensory lateral line lobe (ELL) (Krahe and Maler 2014). There firing rates are drastically decreased to around 50 Hz (Bastian et al. 2002). How such high-frequency information is represented there is not yet understood. Previous accounts show that the encoding scheme changes from the sensory afferents to the next stage of neuronal processing in the brain: The afferents are particularly good encoders of the stimulus time-course while the postsynaptic pyramidal neurons show a better performance in stimulus discrimination and feature extraction, and have better signal-to-noise ratios than P-units (Gabbiani et al. 1996).

### The stochastic LIF model confirms and defies intuition

It makes intuitive sense that the representation of the dynamic stimulus depends on the spike rate. Signaling fast modulations in the stimulus requires more spikes than signaling slow modulations. Our result confirms this intuition as the ICF grows approximately linearly with increasing firing rate, though it remains well below the firing rate (bisecting line) for firing rates above approximately 100 Hz for small $\tau _m$. Finding this behavior in such a basic integrate-and-fire model indicates that it is a generic property of spiking neurons. Towards lower firing rates, however, the ICF remains constant over a range of firing rates in the case of low noise (D=0.001, Fig. 3B, D). At even lower firing rates (below approx. 100 Hz for small $\tau _m$ and 50 Hz for higher $\tau _m$, Fig. 3B, D), the ICF in the LIF model can substantially exceed the firing rate. Moreover, in this low-rate range it depends only on the membrane time constant, Eq. (22). This is an unexpected effect we do not observe in the P-units that display only significantly higher firing rates.

**Fig. 6:**
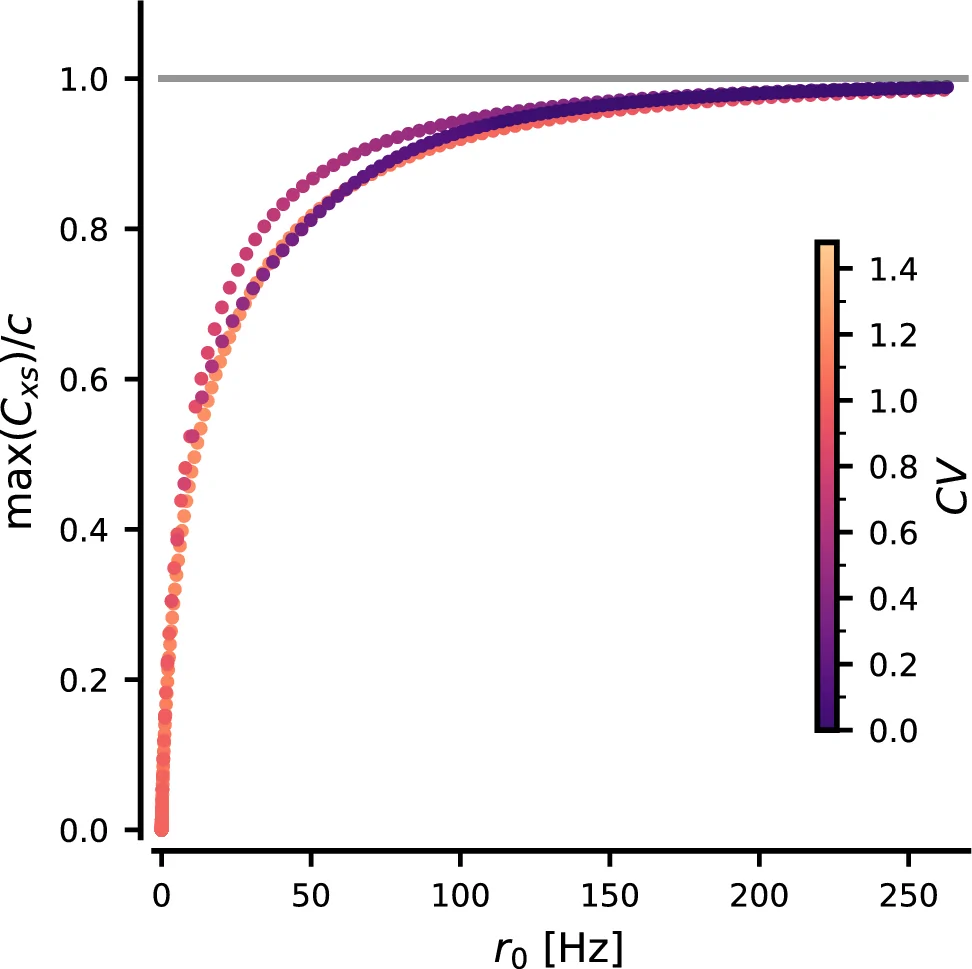
Maximum of the coherence function ($C_{xs}(0)$) for the LIF model Eq. (12) plotted against firing rate. Data points are colored according to the *CV* that corresponds to the chosen (μ, *D*). Grey line indicates upper bound of the coherence function that is given by construction. Here, $\tau _m =$ 10 ms and $\tau _\text {ref}=$ 0

**Fig. 7:**
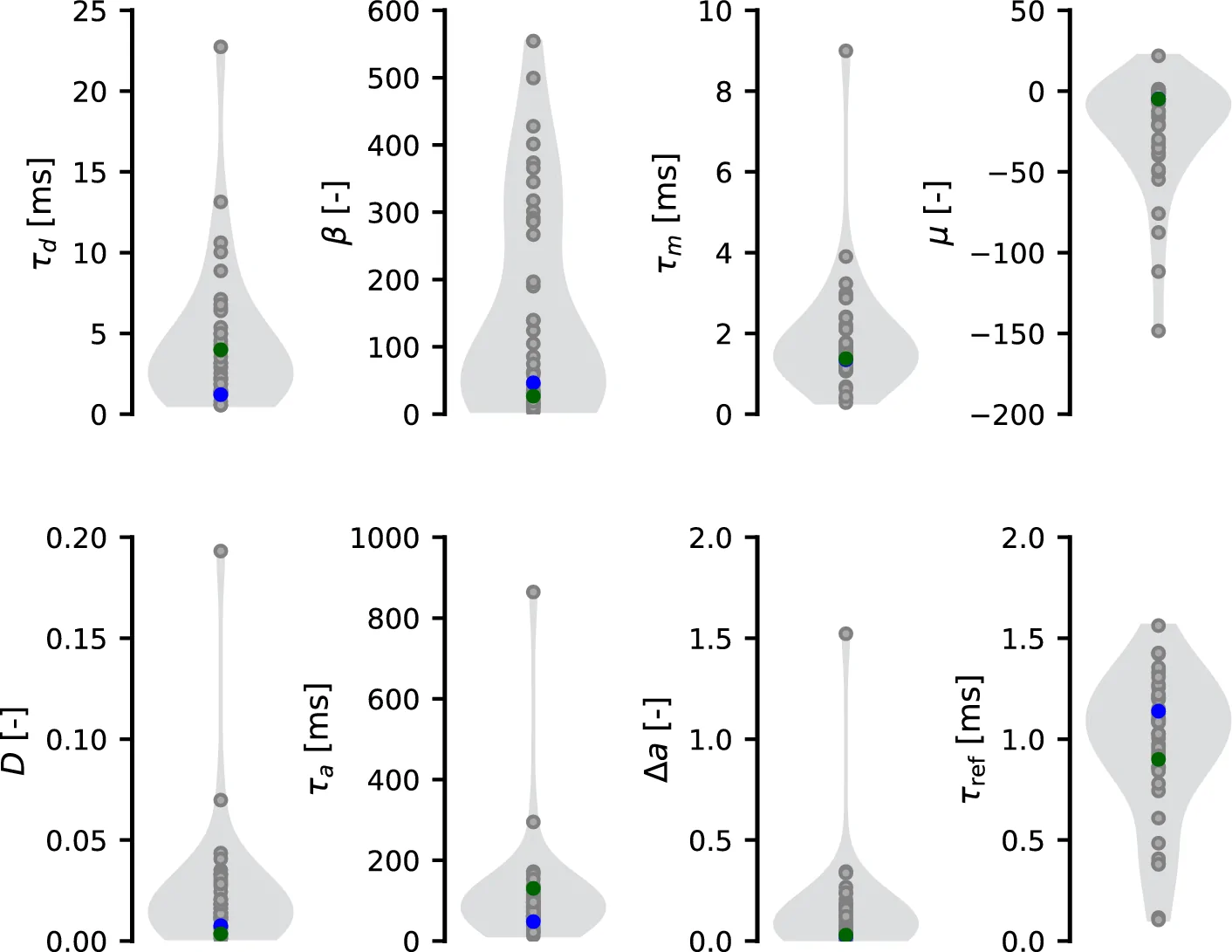
Distributions the model parameters among the 39 model neurons (see methods Sect. 2.2). Green and blue colored dots highlight the example model cells depicted in Fig. 2 panel C

While the ICF remains far above the firing rate, its value is still low in absolute terms. Together with the drastic decrease of the coherence function with decreasing firing rate (Fig. 3E, Fig. 6), the ability of a neuron to transmit high-frequency information with a low spike rate is, thus, severely limited.

### The ICF as a measure of high-frequency information transmission

The ICF is by definition, Eq. (2), first and foremost a measure of the width of the coherence function. It contains no information about the amplitude of the coherence function and is only based on two values. It thus should be used with caution to judge the information transmission at high frequencies.

As an alternative to the ICF one could consider a measure based on the lower-bound formula of the mutual information rate, Eq. (3), taking more than just two values of the coherence function into account. One could i) choose a fixed ’high’ frequency $f_\textrm{i}$ and calculate how much of the total mutual information rate originates from frequencies higher than $f_\textrm{i}$

23 $$\begin{aligned} R_{\text {Info, }f_\textrm{i}} = - \int _{f_\textrm{i}}^\infty \textrm{d}f\, \log _{2}\left[ 1-C_{xs}(f)\right] , \end{aligned}$$

or ii) find a frequency $f_\textrm{ii}$ such that integrating up to said frequency gives a fixed fraction γ of the total mutual information rate

24 $$\begin{aligned} \gamma = \frac{-\int _{0}^{f_\textrm{ii}} \textrm{d}f\, \log _{2}\left[ 1-C_{xs}(f)\right] }{R_\text {Info, LB}} \end{aligned}$$

The integral in the numerator could then also be used to judge the total information transmission up to $f_\textrm{ii}$.

However, these alternatives have a common disadvantage. Both rely on integrating the coherence function which is only available over a limited frequency range when estimated from data. This frequency range is an additional arbitrary parameter that can hinder comparisons between different experimental and simulation setups. We therefore prefer the combination of the ICF as a measure of the coding bandwidth with the maximum of the coherence function (see Fig. 6, Appendix 1) as a measure of the magnitude of the coherence function to judge the information transmission at high frequencies.

### Coding bandwidth of non-monotonic coherence functions

In general, coherence functions can feature more complex frequency dependencies than the monotonic decrease mostly observed in this study. For instance, driving the LIF model with colored noise or subthreshold oscillations can lead to a high-pass or band-pass character of coherence function (Lindner 2016). In such cases, the ICF might not provide a satisfying estimate of the coding bandwidth. Therefore, we here put forward an alternative to measuring the coding bandwidth using the ICF.

Let $\mathcal {A}$ be the set of all positive frequencies at which the coherence function exceeds half of its maximum, i.e. of the threshold-reset distance and with respect to the reset voltage as a reference point; this choice implies $v_T$ and $v_R$ being set to unity and zero, respectively.

25 $$\begin{aligned} \mathcal {A} = \{f\in \mathbb {R}^+:C_{xs}(f)>1/2\max C_{xs}\}. \end{aligned}$$

Then, one can obtain the coding bandwidth by summing up all frequencies contained in $\mathcal {A}$:

26 $$\begin{aligned} CBW = \int _\mathcal {A} \textrm{d}f. \end{aligned}$$

Note that for low-pass coherence functions Eq. (26) yields the ICF.

### General validity of the observations

In the electrosensory system we do have sensory afferents with rather high firing rates. Further, high-frequency signals were found to be of particular behavioral importance during courtship. Still, our results are not limited to the electrosensory system. Rather, the observations are of general applicability. In particular, the analytical derivations of the ICF in the stochastic leaky-integrate and fire model show that a reduction in firing rate, down to very low firing rates of a few spikes per second, always goes along with a reduced ICF. That is, any (sensory) system that needs to encode information about fast changes in the input should employ neurons with appropriate firing rates. Conversely, a system that does not need to encode such fast signals may benefit from slower-firing neurons to reduce the energetic costs of neuronal encoding (Sterling and Laughlin 2015).

This relationship may, however, be modulated by short-term synaptic plasticity. In these high-firing neurons, synaptic depression can attenuate high-frequency signals, even though the role of depression and facilitation in information filtering is not always intuitive. Both forms of short-term plasticity may leave the information transmission unchanged in certain situations (Lindner et al. 2009), short-term synaptic depression may lead to a high-pass filtering of information if noise in the transmitter-uptake is taken into account (Rosenbaum et al. 2012) or in situations with synaptic heterogeneity and several input signals (Droste et al. 2013). The proposed principle should be applicable to any processing stage and any system insofar as their function is to *densely* encode the time-course of the input signal. Different consideration may apply when the system employs other coding strategies, such as synchrony across neurons in a population.

## Conclusion

In this work we demonstrated that the information-cutoff frequency (ICF) depends on the firing rate. Neurons with high firing rates can encode faster stimulus dynamics than cells with low firing rates. We observed this relationship in experimental data and model simulations based on leaky integrate-and-fire neurons. Using the generic stochastic LIF model we can derive analytical expressions linking the ICF to the membrane time-constant and the firing rate. Interestingly, at very low firing rates, this dependency does not hold anymore and the ICF is fully determined by the membrane time constant.

That the ICF decreases with lower firing rates is especially important in the context of decreasing firing rates observed along neural processing pathways from sensory periphery to higher brain areas (Clemens et al. 2011; Vinje and Gallant 2000; Laurent 2002; Sterling and Laughlin 2015). In many species and sensory modalities, subsequent processing involves a separation into ON and OFF channels involving lower firing rates, which is, from an energetic perspective, beneficial (Gjorgjieva et al. 2014, 2019). How the reduction in firing rate is compatible with the relevance of high-frequency information remains unknown.

## Figures and Tables

**Fig. 5:**
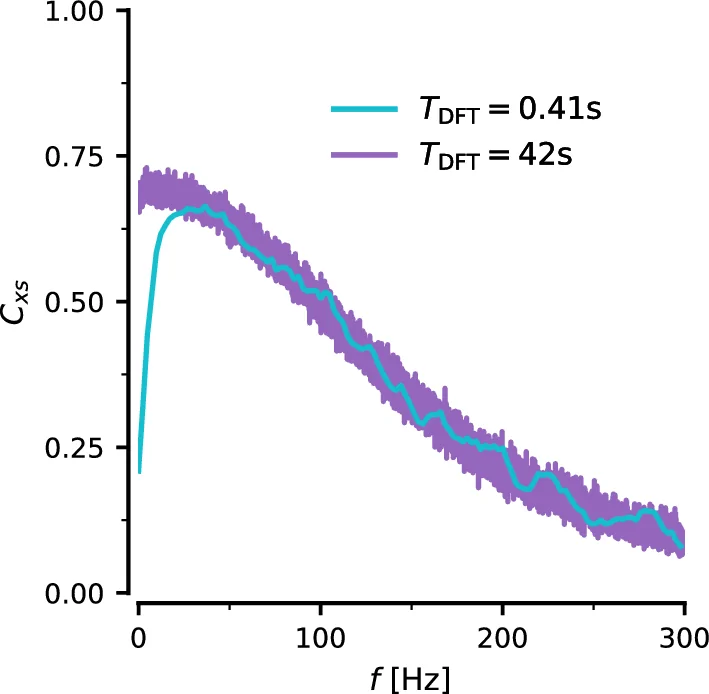
Estimating Fourier spectra on short segments leads to low-frequency coherence drop-off. Coherence functions of the P-unit model neuron when estimating the spectra with different segment lengths ($T_{\text {DFT}}$). The cyan line represents the segment length used for the analysis of experimental data ($T_{\text {DFT}}$ = 0.42 s; Δf = 2.44 Hz) and purple 100 times longer segments of 42 s (Δf = 0.029 Hz). Note: The cyan spectrum appears less noisy which is a consequence of the frequency binning

## Data Availability

The used experimental data is available under the following DOI: https://doi.gin.g-node.org/10.12751/g-node.5b08du Analysis code is available upon request.

## A Short segment lengths lead to low-frequency coherence drop-off

The coherence functions of the experimentally recorded and the simulated P-units deviate in the low-frequency regime (<50 Hz). The experimental data shows a strong drop-off towards low frequencies that is not present in the simulations (Fig. 2). The adaptation current, that is present in both real and simulated P-units can not explain the difference. It acts as a high-pass filter on the transfer function attenuating low-frequency responses (Benda and Herz 2003; Fisch et al. 2012) but it also attenuates low-frequency components of the noise (Middleton et al. 2003) and leaves the SNR, and thus the coherence, in these frequency bands unaffected.

In order to resolve the discrepancy, we used the P-unit model and analyzed the stimulus–response coherence in two ways: (i) similar to how experimental data were analyzed estimating the Fourier spectra with relatively short data segments ($2^{13}$ data points at 20 kHz sampling, i.e. $T_{\text {DFT}}$ = 0.42 s), and (ii) with long segments of 42 s (purple, Fig. 5). For each condition, 1000 trials were analyzed. The choice of the segment length defines the resolution of the Fourier spectrum and also how well the Fourier components can be estimated.

To better understand the effect of the segment size (or, equivalently, the time window $T_{\text {DFT}}$ of the Fourier transform), we consider specifically the spike-train power spectrum which forms the nontrivial part of the denominator in the coherence function (see Eq. (9)). The power spectrum at small frequencies can be related to the Fano factor, $F(T)=\langle (N(T)-\langle N(T)\rangle )^2\rangle /\langle N(T)\rangle$ (the spike count’s variance divided by its mean value), or, equivalently, to the interspike interval’s coefficient of variation, *CV* and the sum over the serial correlation coefficients $\rho _k=\langle I_{i+k}I_i-\langle {I_i}^2\rangle \rangle /\langle I_{i}^2-\langle {I_i}^2\rangle \rangle$ (McFadden 1962):

A1 $$\begin{aligned} \lim \limits _{f\rightarrow 0}S(f)=\lim \limits _{T\rightarrow \infty }F(T)={CV}^2\left( 1+2\sum _{k=1}^\infty \rho _k\right) . \end{aligned}$$

Interestingly, for the strongly adapting P-units the sum of the serial correlation coefficients is close to -1/2 (Ratnam and Nelson 2000), resulting in a reduction of the Fano factor (Chacron et al. 2001) and, consequently, strongly diminished noise power at low frequencies (Chacron et al. 2004a, b). Negative correlations among the interspike intervals (ISIs) make the number of spikes *N*(*T*) in a time window $T_{\text {DFT}}$ much more reliable than would be expected given the variability of the single ISI (an intuition that is based on the tacit assumption of a renewal process, i.e., a point process with independent ISIs). However, this holds only for a sufficiently long $T_{\text {DFT}}$ – if the time window $T_{\text {DFT}}$ is not ’long enough’, the full noise-shaping effect of the negative serial correlations has not been achieved yet: counts are still more variable because we essentially average over realizations of the stochastic process with different values of the adaptation variable that do not equilibrate during the time window. The corresponding finite-time-window estimate of the spike-train power spectrum has still more power at low frequencies than the true spectrum (i.e. the one obtained in the long-time limit).

The high-pass filtering of the spike train’s mean value which makes up the numerator in the coherence function according to Eq. (9) does not have a similarly sensitive dependence on the time window of the Fourier transform. Hence, the coherence function at low frequencies is small because the system has ’too much’ noise power at low frequencies when the time window or segment $T_{\text {DFT}}$ is too short. The relevant scale is here obviously the slowest time scale in the system, namely, the adaptation time constant. We may expect that the power spectrum and thus the coherence function become independent on the segment length once $T_{\text {DFT}}\gg \tau _\text {a}$. From the numbers given above, it is clear that the condition is met for a segment length of 42 s, which is indeed much larger than the adaptation time constant of $\tau _a=71.4$ ms ($\tau _a$ in Eq. (19), median: 83.4 ms inter-quartile range: 60.1 ms to 123.7 ms).

## B Coherence function in the rare-spiking regime

To investigate the rare-spiking regime, we first have to translate $r_0\rightarrow 0$ into the parameters of the LIF model μ and *D*. There are two ways to this. The first intuitive choice might be to turn down the mean input ($\mu \rightarrow -\infty$) but this leads to more involved calculations. Instead, we consider intermediate μ, namely $v_R< \mu <v_T$, with vanishing noise (D→0). In other words, we reduce the noise in the noise-driven/excitable regime making spiking progressively less likely.

With μ and *D* set, we can now turn to the expression for the coherence function in terms of parabolic cylinder functions, Eq. (12). For the arguments of the parabolic cylinder functions $z_{T/R} = (\mu - v_{T/R})/ \sqrt{D}$, our chosen setting corresponds to $z_R\rightarrow \infty$ and $z_T \rightarrow -\infty$. In this case, the asymptotic ti of the parabolic cylinder functions have power-law from National Institute of Standards and Technology (2025).

In the following, we switch to the notation of the parabolic cylinder functions used in (National Institute of Standards and Technology 2025):

A2 $$\begin{aligned} \mathcal {D}_{a}(z) = U(-a-1/2, z) \end{aligned}$$

and define $\omega = 2\pi f \tau _m$ to keep the expressions more concise. We start by considering the $z_R$ terms

A3 $$\begin{aligned} A_1 =&\, \textrm{e}^{\frac{z_R^2}{4}} U\left( \frac{1}{2}-i\omega , z_R \right) \end{aligned}$$

A4 $$\begin{aligned} A_2 =&\, \textrm{e}^{\frac{z_R^2}{4}} U\left( -\frac{1}{2}-i\omega , z_R \right) . \end{aligned}$$

For both expressions we can use relation (12.9.1) from National Institute of Standards and Technology (2025)

A5 $$\begin{aligned} U(a,z)\sim \textrm{e}^{-\frac{z^2}{4}}z^{-a-\frac{1}{2}}\sum _{s=0}^{\infty } (-1)^s \frac{(\frac{1}{2} + a)_{2s}}{s!(2z^2)^s}. \end{aligned}$$

Applying it, we obtain in leading order in $z_R$ (s=0)

A6 $$\begin{aligned} A_1 \sim&\, \frac{z_R^{i\omega }}{z_R} \end{aligned}$$

A7 $$\begin{aligned} A_2 \sim&\, z_R^{i\omega }. \end{aligned}$$

We see that $A_1$ vanishes for $z_R \rightarrow \infty$.

The $$z_T$$ terms

A8 $$\begin{aligned} A_3 =&\, \textrm{e}^{\frac{z_T^2}{4}} U\left( \frac{1}{2}-i\omega , z_T \right) \end{aligned}$$

A9 $$\begin{aligned} A_4 =&\, \textrm{e}^{\frac{z_T^2}{4}} U\left( -\frac{1}{2}-i\omega , z_T \right) . \end{aligned}$$

require additional steps as $z_T$ tends to -∞. Here, we first apply a relation called connection formula ((12.2.15) from National Institute of Standards and Technology (2025)) to invert the second argument of the parabolic cylinder function

A10 $$\begin{aligned} U(a,-z) = -\sin (\pi a)U(a,z) + \frac{\pi }{\Gamma \left( \frac{1}{2}+a\right) }V(a,z) \end{aligned}$$

at the cost of introducing the other parabolic cylinder function *V*(*a*, *z*) (parabolic cylinder function come in pairs of two as they are solutions of a second order ODE). There also exists a formula for the asymptotics of *V*(*a*, *z*) for $z\rightarrow \infty$ ((12.9.4) from National Institute of Standards and Technology (2025)):

A11 $$\begin{aligned} V(a,z)\sim \sqrt{\frac{2}{\pi }} \textrm{e}^{\frac{z^2}{4}}z^{a-\frac{1}{2}}\sum _{s=0}^{\infty } (-1)^s \frac{(\frac{1}{2} - a)_{2s}}{s!(2z^2)^s}. \end{aligned}$$

Applying Eq. (A10) to $A_3$ and $A_4$ followed by using Eqs. (A5) and (A11) to expand *U* and *V*, respectively, we obtain

A12 $$\begin{aligned} A_3 \sim&\, \sin \left( \frac{\pi }{2} - i\omega \pi \right) \frac{(-z_T)^{i\omega }}{z_T} + \frac{\sqrt{2\pi }\,\textrm{e}^{\frac{z_T^2}{2}}}{\Gamma (1-i\omega )(-z_T)^{i\omega }}\end{aligned}$$

A13 $$\begin{aligned} A_4 \sim&\, \sin \left( \frac{\pi }{2} + i\omega \pi \right) (-z_T)^{i\omega } - \frac{\sqrt{2\pi }\, \textrm{e}^{\frac{z_T^2}{2}}}{\Gamma (-i\omega )z_T(-z_T)^{i\omega }}. \end{aligned}$$

Since $|z_T|\gg 1$, we can omit the first term in both Eqs. (A12) and (A13):

A14 $$\begin{aligned} A_3 \sim&\, \frac{\sqrt{2\pi }\,\textrm{e}^{\frac{z_T^2}{2}}}{\Gamma (1-i\omega )(-z_T)^{i\omega }}\end{aligned}$$

A15 $$\begin{aligned} A_4 \sim&\, -\frac{\sqrt{2\pi }\, \textrm{e}^{\frac{z_T^2}{2}}}{\Gamma (-i\omega )z_T(-z_T)^{i\omega }}. \end{aligned}$$

We can now insert $A_1$ to $A_4$ back into Eq. (12)

A16 $$\begin{aligned} C_{xs}(\omega ) =&\, \frac{2c\tau _m r_0 \omega ^2}{1+\omega ^2} \frac{|A_3-A_1|^2}{|A_4|^2-|A_2|^2}\end{aligned}$$

A17 $$\begin{aligned} \sim&\, \frac{2c\tau _m r_0 \omega ^2 z_T^2}{1+\omega ^2} \left| \frac{\Gamma (-i\omega )}{\Gamma (1-i\omega )}\right| ^2, \end{aligned}$$

where we used $|z_T^{i\omega }|^2 = 1$, and again that $|z_T|$ is large. As a final step, we use the basic property of the gamma function $\Gamma (x+1) = x\Gamma (x)$. By doing so, we arrive at the asymptoic behavior of the coherence function in the case of rare firing

A18 $$\begin{aligned} C_{xs}(\omega ) \sim \frac{2c\tau _m r_0 z_T^2}{1+\omega ^2}. \end{aligned}$$

The cutoff frequency of this Lorentzian curve is the solution of

A19 $$\begin{aligned} c\tau _m r_0 z_T^2 = \frac{2c\tau _m r_0 z_T^2}{1+\omega ^2_\textrm{cutoff}}, \end{aligned}$$

which is $\omega _\textrm{cutoff} = 1$. Substituting $2\pi f\tau _m$ for ω yields the results in Eqs. (22) and (21).

## C Maximum of the coherence function and firing rate

The actual information transmission characterized by the coherence function is not only determined by the frequency at which the coherence function attains half of its maximal value but of course the absolute value of said maximum matters as well. Therefore, we also studied how the maximum of the coherence function depends on the firing rate. For the LIF model, this is straightforward as the maximum is always at f=0 and we are in full control of the model parameters.

Figure 6 shows the relation between the maximum of the coherence function and the firing rate for the LIF model ($D \in \{0.01, 0.1,1\}$ and various μ). The maximum tends to zero for rare spiking unlike the cutoff frequency which stays at a finite value. Initially, the maximum of the coherence function grows rapidly with increasing firing rate before tending to the optimal value of 1. This general trend is observed for all noise intensities we investigated.

Only for intermediate firing rates, we see deviations based on the fluctuations of the spiking. Here, for a fixed firing rate, the maximum of the coherence function depends non-monotonically on the *CV*. There is a optimal amount of fluctuations in the output of the neuron for maximizing the coherence at f=0. An unexpected effect not observed for the cutoff frequency that is reminiscent of stochastic resonance (Gammaitoni et al. 1998; McDonnell and Ward 2011).

## D Parameter distributions of P-unit model neurons

Model parameters were fitted using a simplex-fit algorithm to the experimentally recorded cells. The distributions in Fig. 7 illustrate the spread for all eight parameters. In particular the parameter β, i.e. the input scaling, varies considerably reflecting the large heterogeneity among P-units. The colored dots show the parameters of the two featured model cells in Fig. 2. It can be noted that the model parameters for these model cells are always in the bulk of the distributions, i.e. they can are typical, not extreme variants of their kind.
